# The influence of leadership on employees' employability: a bibliometric analysis, systematic literature review, and research agenda

**DOI:** 10.3389/fpsyg.2023.1092865

**Published:** 2023-06-02

**Authors:** Joost Hoedemakers, Arne Vanderstukken, Jol Stoffers

**Affiliations:** ^1^Faculty of Management, Open Universiteit, Heerlen, Netherlands; ^2^Research Centre for Employability, Zuyd University of Applied Sciences, Sittard, Netherlands; ^3^Research Centre for Education and the Labour Market (ROA), Maastricht University, Maastricht, Netherlands

**Keywords:** employability, leadership, social exchange theory, supervisors, employees, systematic literature review

## Abstract

**Introduction:**

Policymakers, researchers, and practitioners have recently begun treating employability-an individual's ability to possess and continuously adjust and acquire up-to-date competencies, flexibility, adaptability, and openness to change-as crucial to enabling employees to respond to ubiquitous and rapid changes in organizations (e.g., changing tasks and work-related processes). Research into ways to enhance employability, particularly through supervisor leadership, which, for example, facilitates training and competence development, has thus grown in popularity. A review on leadership as an antecedent of employability is both evident and timely. This review thus addresses the question of whether a supervisor's leadership influences employees' employability, and in which contexts and through which mechanisms it does so.

**Methods:**

As preliminary study we conducted a bibliometric analysis (which corroborated employability's recent rise in popularity) and as main study we conducted a systematic literature review. For this, the authors independently searched for articles, which met the inclusion criteria and subsequently were included for full text analysis. The authors also independently used the forward and backward snowballing technique for identifying additional articles which met the inclusion criteria and subsequently were included for full text analysis. The procedure resulted in 17 articles in total.

**Results:**

Most of the articles identified positive relationships among several conceptualizations of supervisor leadership and employee employability, such as transformational leadership and leader-member exchange, and to a lesser extent, servant leadership and perceived supervisor support. This review suggests that such relationships occur across different work contexts, such as educational, SMEs, healthcare, and several other industries, and these contexts also vary geographically.

**Discussion:**

The relationships among supervisor leadership and employee employability are largely explained using a social exchange perspective, which means that the positive influence of leadership on employability is itself influenced by a two-way social exchange relationship between supervisor and employees. The quality of the dyadic relationship between leader and followers thus determines the extent to which leaders offer valuable resources such as training and feedback, which subsequently enhances employees' employability. This review demonstrates that investing in supervisors' leadership is a valuable HRM strategy that fosters employability, and it identifies practical implications that inform policy and practice and sets an agenda for future employability research.

## 1. Introduction

Employability, defined as the ability to adjust and acquire up-to-date competencies, and being flexible, adaptable, and open for change (Van der Heijden et al., 2018; Van Harten et al., 2020), has recently attracted policymakers', researchers', and practitioners' attention (OECD, 2019). Employability allows employees to respond to contemporary ubiquitous and rapid changes in organizational environments (e.g., changing tasks and work-related processes; Bozionelos et al., 2016), thus changing job demands. Worldwide megatrends cause various rapid changes, such as ongoing technological innovation (Baptista et al., 2020; Henderikx and Stoffers, 2022), hyper-competition (D'Aveni, 1994), aging of the populations, and the COVID-19 pandemic (Rudolph et al., 2020). For example, COVID-19 influenced work-related processes, and employees had to subsequently cope with work-family challenges because work became increasingly organized due to working from home, telecommuting, and virtual teamwork (Rudolph et al., 2020).

Employability helps employees cope with rapidly changing job demands for two reasons. First, employable employees are, by definition, more skilled and flexible than those who are less employable; they climatize quickly to, and even thrive in, new environments (Van der Heijden et al., 2018). Second, employable employees are less likely to develop feelings of job insecurity and panic in reaction to changes; they are confident that they can pursue employment, outside of the current organization if necessary (De Cuyper and De Witte, 2011). Employability contributes to optimal employee functioning (Vanhercke et al., 2014), which subsequently enhances organizational success, for example, in terms of performance (Camps and Rodríguez, 2011) and lower turnover intentions (Nauta et al., 2009). Employability is thus crucial to contemporary employees and employers.

Historical analyses suggest that employability associates with transitions in both the labor market and organizations (Thijssen et al., 2008). Over time, the meaning of employability evolved, resulting in multiple conceptualizations (Forrier and Sels, 2003). The current systematic literature review uses an input-based approach of employability (Vanhercke et al., 2014), focusing on “the subjective perception held by an employee (or by his or her supervisor) about his or her possibilities in terms of competences, to obtain and maintain work” (Van der Heijden et al., 2018, p. 237). Drawing from conceptualizations of employability in human resources management (HRM) and career psychology literature (Fugate et al., 2021), we argue that employability manifests in employer-employee relationships, with employers (e.g., HRM professionals and supervisors) and employees as focal stakeholders. The individual challenge of retaining and enhancing employability is a shared responsibility among employers and employees, and organizations must thus be adaptable since employers are unable to guarantee lifetime job security. Employers focus on employability because it enhances agreements between employers and employees—that is, the psychological contract (Garavan, 1999)—and, as a result, such contracts motivate employees to agreements in the employer-employee relationship (Rousseau, 2004).

The pandemic transformed job demands (e.g., increasement of remote working), consequently employers focus on job resources such as leadership to empower employees so that they are able to respond to challenging post-pandemic job demands (Manuti et al., 2022). Employers seek HRM strategies that enhance employability (Veth et al., 2018), and using such strategies, they facilitate job resources, such as leadership (e.g., contexts), so employees are able to cope with job demands, including, for example, work overload (Bakker and Demerouti, 2017). Employability as a dual employer-employee responsibility namely suggests an increase of the resources of the organization, which enhances the competitive advantage at the organizational level (Vermeeren and Van der Heijden, 2022), and provides career perpective at the individual level (Van der Heijde et al., 2018). However, despite the obvious benefits of employability to organizations, some employers remain reluctant to invest in employability. De Cuyper and De Witte (2011) evidence the employability paradox, which demonstrates risks to organizations, such as increased turnover intentions. The question, then, is which HRM strategies foster employability. Extant studies suggest that job resources (Bakker and Demerouti, 2017), such as training and development opportunities (Van der Heijden et al., 2009; Froehlich et al., 2015) and job's learning value (Le Blanc et al., 2019), foster employees' employability. Several authors thus stress the urgency of “learning to become employable” (Houben et al., 2019, p. 1). Similarly, supervisors' leadership, as a work-related, contextual factor (e.g., a job resource), also stimulates employability (Clarke and Patrickson, 2008; Van der Heijden and Spurk, 2019), for example, by empowering followers and facilitating training and competence development (Becker, 1962). Such leadership, as both context and determinant, might contribute to employability (Van der Heijde and Van der Heijden, 2014).

From a relational perspective, leadership associates with “a social influence process through which emergent coordination (i.e., evolving social order) and change (e.g., new values, attitudes, approaches, behaviors, and ideologies) are constructed and produced” (Uhl-Bien, 2006, p. 655). This is particularly true because the influences of training and development on employees' employability are also influenced by both leadership and the quality of the relationship between supervisors (e.g., employer or leader) and the employee (Struzyna and Marzec, 2017). From an employer-employee relationship perspective, better understanding of the leadership-employability relationship is paramount (Fugate et al., 2021), especially when talent is scarce and retraining employees is important to employers, and when investing in employability is crucial. A literature review of supervisors' leadership as antecedents to employees' employability enhancement is thus evidently needed (Chughtai, 2019; Wang et al., 2019).

To advance employability research, this review identifies, selects, and evaluates extant studies to report on the state of knowledge (Denyer and Tranfield, 2009) regarding the relationship between supervisors' leadership and employees' employability. To address high-quality reporting, which is “transparent, complete and accurate” (8), this review uses Page et al. (2021)'s Preferred Reporting Items for Systematic reviews and Meta-Analyses (PRISMA) to address the research question (Counsell, 1997) of which work contexts and mechanisms influence supervisors' leadership's influence on employees' employability. This review has the potential to inform policy, practice, and research in management and organization studies (Denyer and Tranfield, 2009), especially those related to employability. From both career (i.e., employees) and HRM (employers) perspectives, it is important to synthesize the findings in extant studies to assess for consistency (Petticrew, 2001). This literature review is first to synthesize empirical findings on relationships between supervisors' leadership and employees' employability, and it therefore represents a valuable contribution to employability literature.

## 2. Overview of studies

A triangulation approach was used to enhance the consistency of findings, and subsequently to increase the review's validity (Saunders et al., 2012). We, therefore, began with explorative quantitively bibliometric analysis. We retrospectively report on publication-related metric total publications (TP) on two topics—employability and both leadership and employability. We then map these topics as they relate to employability, and we are thus able to identify research gaps in the literature (Donthu et al., 2021). Research gaps are relevant as a starting point when reporting on the current state of research on a topic in the form of a literature review, which additionally includes recommendations for future research. As the main study, a qualitative systematic literature review (Denyer and Tranfield, 2009) is used to search for and synthesize peer-reviewed Dutch and English studies systematically and subsequently report on the present state of knowledge on the relationship between leadership and employability. Both methodologies allow a systematical, explicit, and replicable (Fink, 2005) investigation of employability literature.

### 2.1. Preliminary study: bibliometric analysis

#### 2.1.1. Methodology

During December 2021, we began with a manual qualitative literature review (Denyer and Tranfield, 2009), and we used a bibliometric methodology to explore the current state of scientific research on employability, particularly the combination of leadership and employability. The purpose was to quantify Chughtai (2019)'s and Wang et al. (2019)'s arguments of research gaps on the relationship between leadership and employability. Bibliometric methodology mitigates researcher bias (Zupic and Cater, 2015) and is thus relevant as a preliminary study to a literature review. The methodology uses quantitative techniques, including, for example, co-occurrence (i.e., co-word) analysis, to identify and analyze large amounts of bibliometric data from scientific databases, such as Web of Science (Broadus, 1987). It extracts bibliometric data (e.g., keywords, journals, and researchers) from scientific databases and it uses them as inputs to allow a researcher to map bibliometric networks using bibliometric software (e.g., VOSviewer; Van Eck and Waltman, 2014; Donthu et al., 2021). Such networks consist of nodes and edges. Nodes represent bibliometric data (e.g., keywords), which depend on a specific analysis (Liao et al., 2018). For example, co-occurrence networks visualize relationships between keywords, and the size of a node represents the frequency of such occurrences in bibliometric data. Edges represent indications and the strength of relationships between nodes (Van Eck and Waltman, 2014).

To discover such indications and the strength of relationships between nodes in a bibliometric network, VOSviewer uses lines to connect nodes. The thickness of a line and the size of a node represent the strength and the occurrence, or co-occurrences, between nodes (e.g., keywords). As a result, a theme-related node forms a thematic cluster. VOSviewer shows nodes that relate to a thematic cluster using color schemes (Van Eck and Waltman, 2014; Donthu et al., 2021). Bibliometric methodology uses two techniques—performance analysis and science mapping (Donthu et al., 2021). Performance analysis considers contributions to a field from research constituents (e.g., authors, topics, and countries) (Cobo et al., 2011), and science mapping analyzes relationships between research constituents (Baker et al., 2021).

#### 2.1.2. Performance analysis

We conducted performance analysis to assess publication-related metric total publications (TP) retrospectively regarding employability and both leadership and employability. These topics associate with keyword searches of an article's title, abstract, and author. We identified bibliometric data and subsequently extracted and analyzed them as output files of the Web of Science (WoS) database using the search string *topic “employability,” publication years until year 2022, document types “article,” and language “English”* OR “*Dutch.”* We also used the Web of Science bibliographic database, using search string *topic “employability”* AND “*leadership,” publication years until year 2022, document types “article,” and language “English”* OR “*Dutch.”*

#### 2.1.3. Co-occurrence analysis

We applied a core technique—keyword co-occurrence analysis—for science mapping. We visualized relationships between keywords in a co-occurrence network (Van Eck and Waltman, 2014), which is used commonly in management research (Phulwani et al., 2020). Keyword co-occurrence analysis focuses on actual content (i.e., words) in publications, such as author keywords, words in article titles, and abstracts as a unit of analysis. Co-occurrence refers to the degree to which two keywords are included in an article's keywords, title, and abstract (Van Eck and Waltman, 2014). To identify, extract, and analyze bibliometric data, we searched WoS database using the search string *topic “employability,” publication years until year 2022, document types “article,” and language “English”* OR “*Dutch.”* We downloaded the resulting bibliographic data in ^*^.txt format in batches of 1000 publications, which were included for further analysis. To analyze and visualize the bibliometric network, we used data from WoS (e.g., output data) as input data in VOSviewer. We applied all keywords as a unit of analysis and full counts as the counting method to visualize co-occurrence networks of the search string (see **Figure 3**) (Van Eck and Waltman, 2014). We used 20 as the minimum number of occurrences of a keyword as a threshold, and we did not verify selected keywords further.

#### 2.1.4. Results

##### 2.1.4.1. Performance analysis

**Figure 1:**
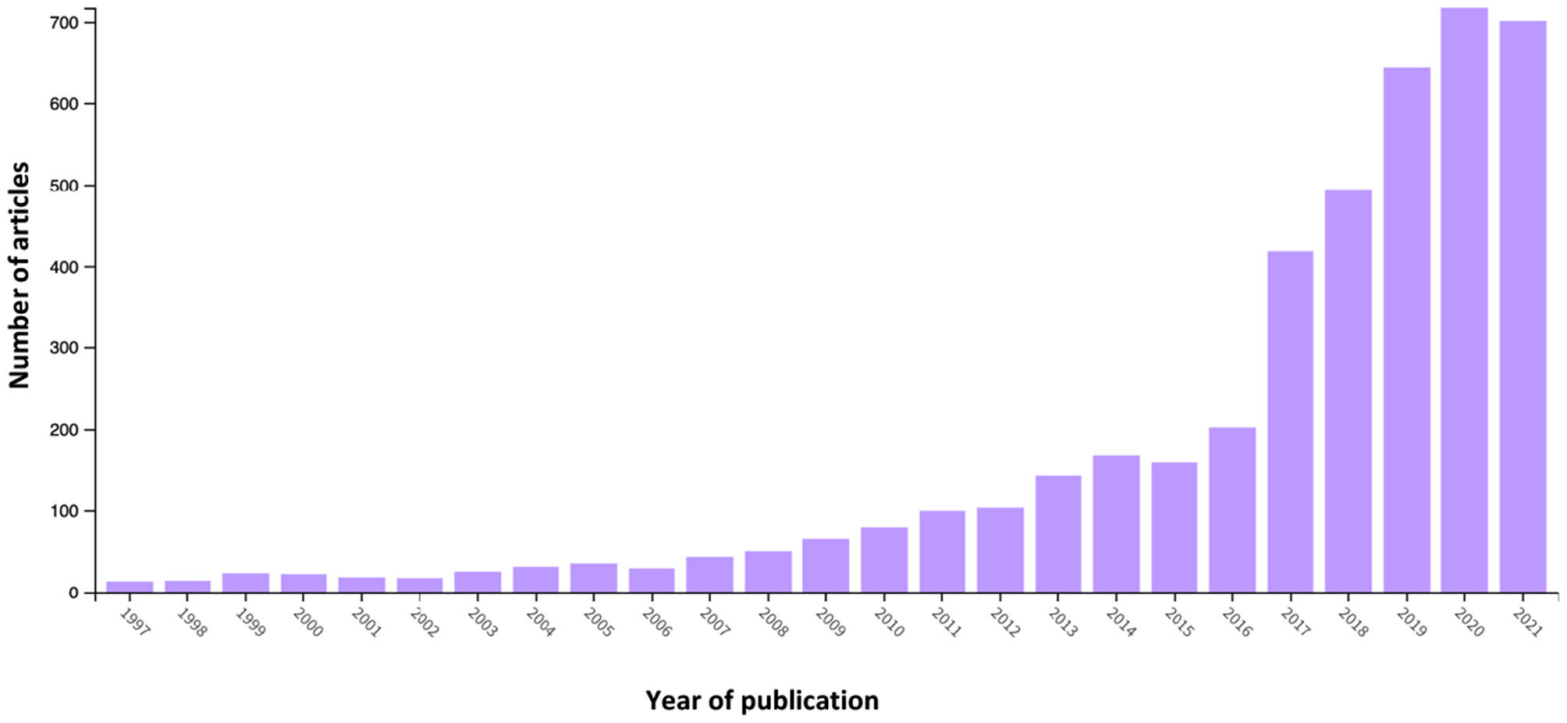
Year-wise number of employability articles (1997–2021). This figure reports the publication trend of scientific articles on the topic of employability to 2022. Data were retrieved from the Web of Science database using the search string: topic “employability,” document type “articles,” language “English” OR “Dutch,” and publication years to 2022.

**Figure 2:**
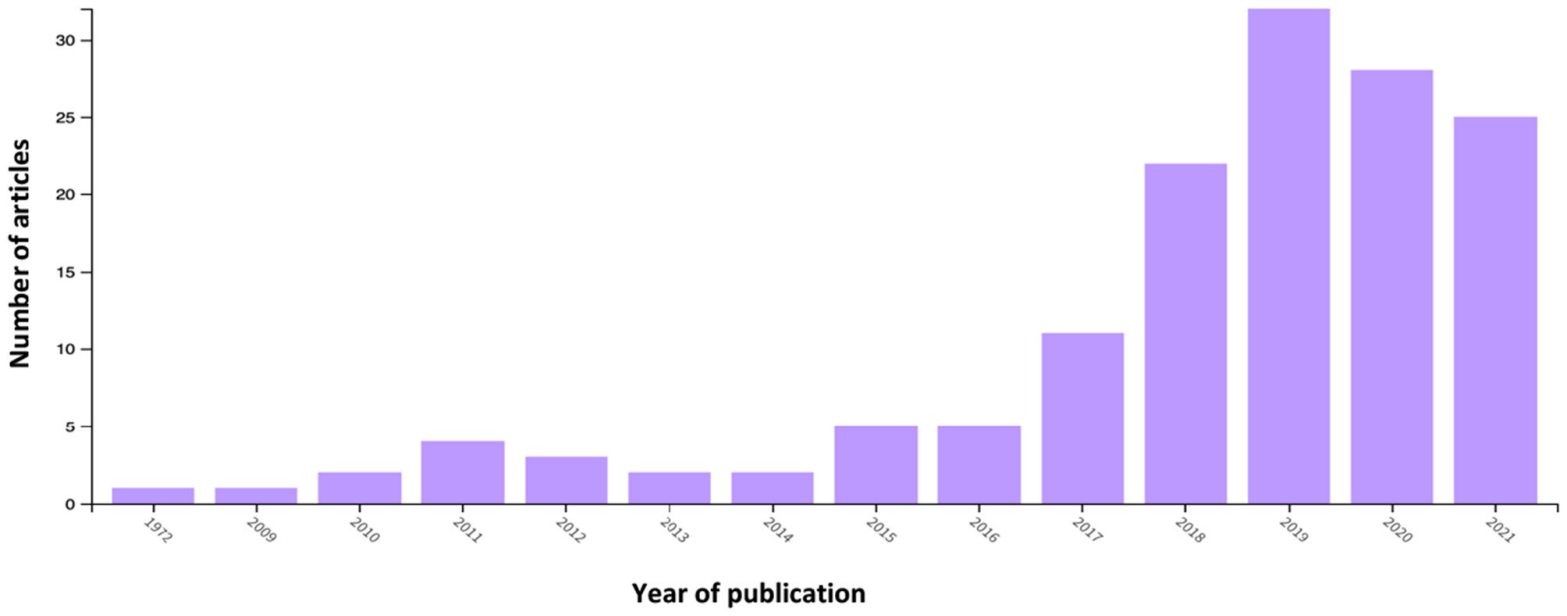
Year-wise number of employability and leadership articles (1972–2021). This figure reports the publication trend of scientific articles on the topics of employability and leadership to 2022. Data were retrieved from the Web of Science database using the search string: topic “employability” AND “leadership,” document type “articles,” language “English” OR “Dutch,” and publication years to 2022.

The search string *topic “employability,” publication years until year 2022, document types “article,” and language “English”* OR “*Dutch,”* resulted in 4453 articles, and search string *topic “employability”* AND “*leadership,” publication years until year 2022, document types “article,” and language “English”* OR “*Dutch,”* resulted in 143 articles. Results suggest that education (34.9%; *n* = 1,556), management (10.4%; *n* = 465), and applied psychology (9.1%; *n* = 405) are the best represented categories in WoS for employability and both leadership and employability (education, 36.4%; *n* = 52; management, 17.5%; *n* = 25; applied psychology, 13.3%; *n* = 19). Outcomes from performance analysis appear in Figures 1, 2, which report publication-related metric total publications (TP) year-wise for both topics (Donthu et al., 2021).

##### 2.1.4.2. Science mapping and co-occurrence analysis

**Figure 3:**
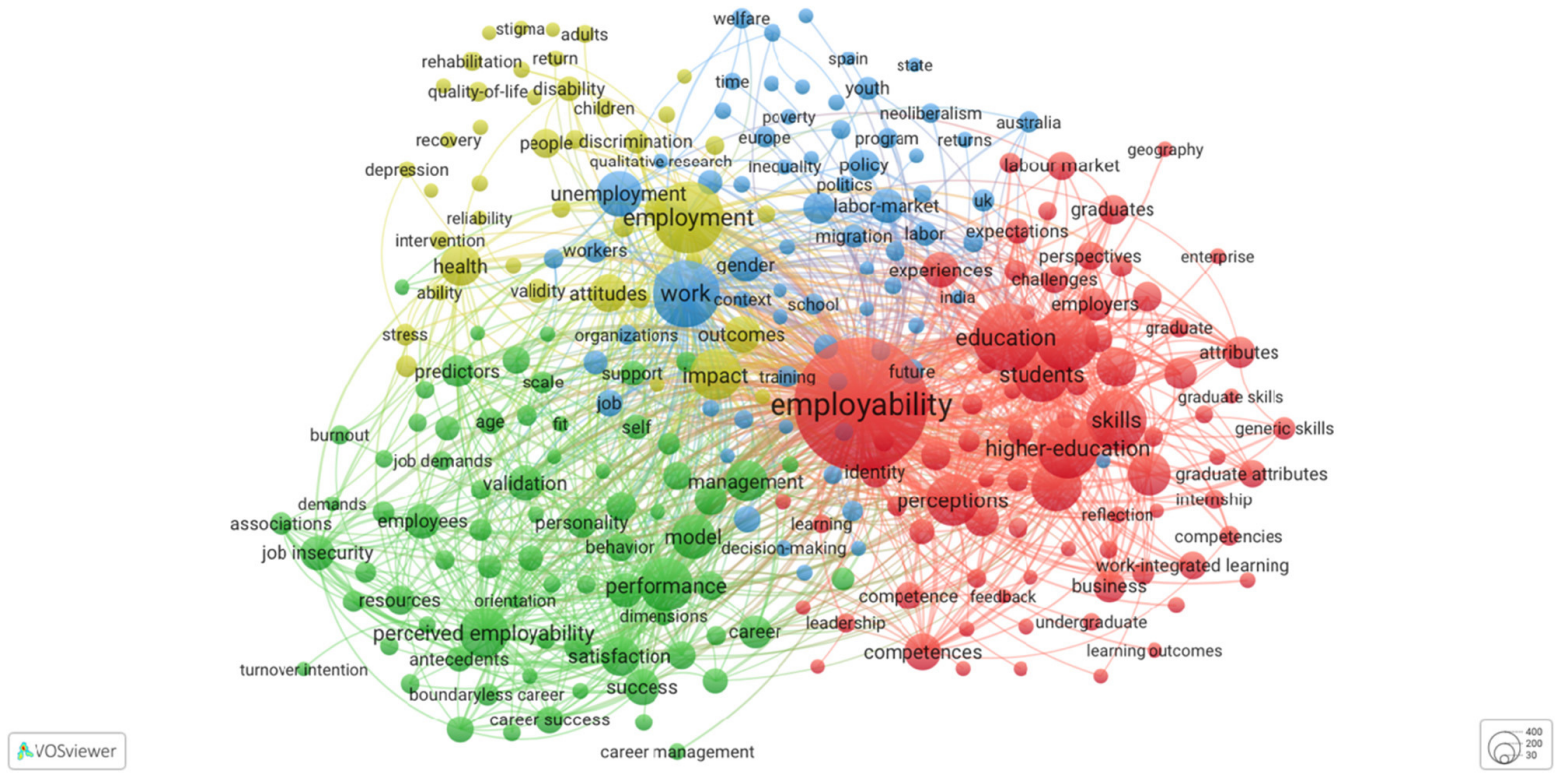
Co-occurrence network visualization using VOS viewer on the topic of employability. This figure reports the publication trend of scientific articles on the topic of employability to 2022. Data were retrieved from the Web of Science database using the search string: topic “employability,” document type “articles,” language “English” OR “Dutch,” and publication years to 2022.

Using VOSviewer, results from co-occurrence analysis on the topic of employability (Figure 3) returned 12,644 keywords, of which 267 met the threshold of 20 as the minimum number of occurrences of a keyword. The 267 keywords were distributed across 4 colored thematic clusters, with 14,134 links and a link strength of 49,482. VOSviewer uses colors to reveal a cluster and links between keywords within a cluster. Results suggest that cluster 1 (red) contained 80 keywords, which focus on education topics (e.g., academic performance and curriculum development), and cluster 2 (green) contained 75 keywords, which associate with work-related topics such as HRM and career development. Cluster 3 (blue) contained 69 keywords related to labor market topics, such as unemployment, and cluster 4 (yellow) contained 43 keywords that focus on health-related topics that relate to employability, such as mental health and depression.

#### 2.1.5. Conclusion

**Figure 4:**
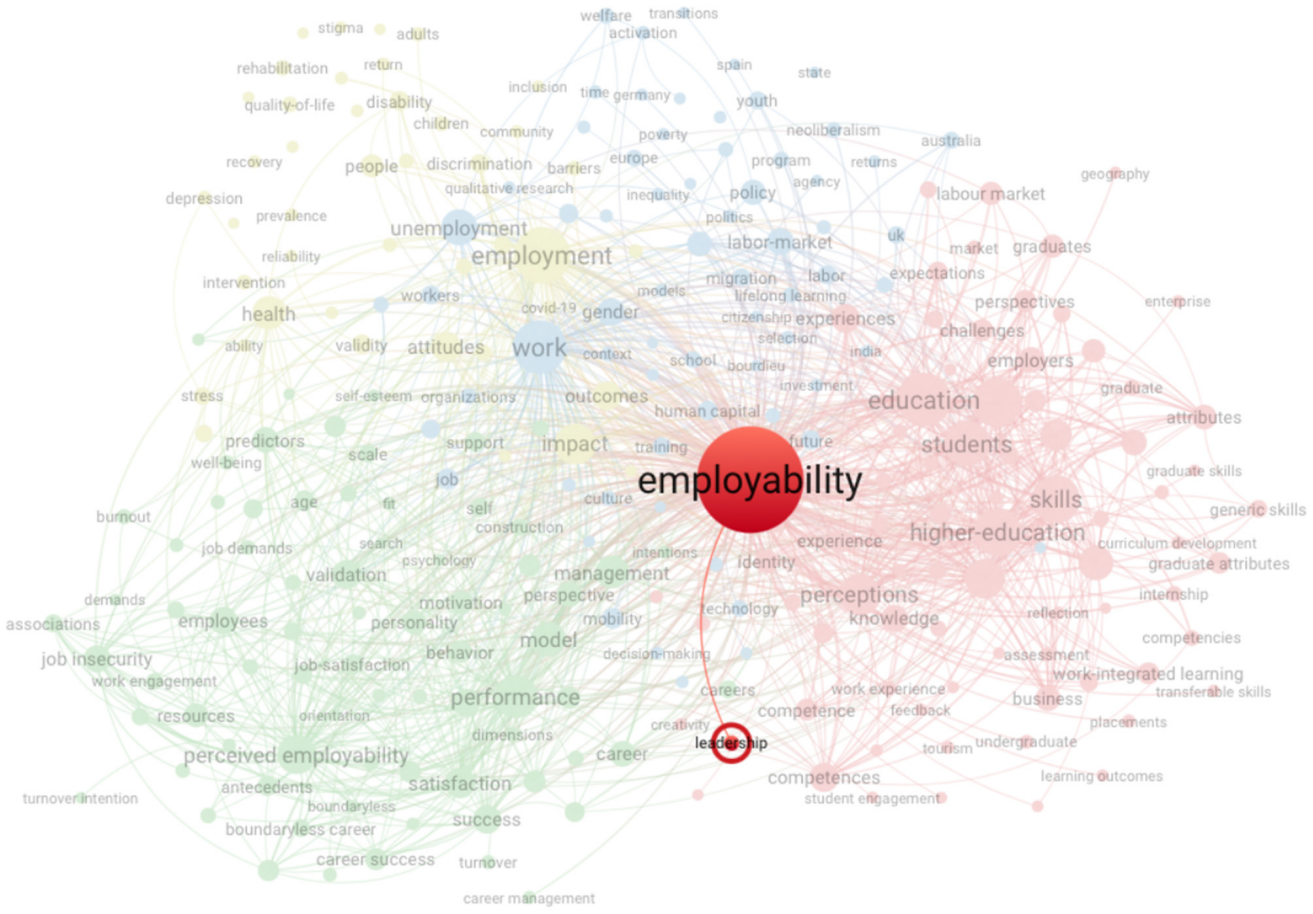
Co-occurrence network visualization using VOS viewer on the topic employability, which revealed the employability-leadership link. This figure reports the publication trend of scientific articles on the topic employability to 2022. Data were retrieved from the Web of Science database using the search string: topic “employability,” document type “articles,” language “English” OR “Dutch,” and publication years 2022.

The performance analysis suggested an increase of research articles on both employability and the combination of employability and leadership, particularly between 2017 and 2022, during which 66.8% (*n* = 2,973) of articles on employability and 82.5% (*n* = 118) on employability and leadership were published. Since 2012, the number of employability articles has exceeded 100. Co-occurrence analysis of keywords suggested that employability links to multiple keywords, including leadership (Figure 4).

Cluster 1 (red), which comprises education and work-related topics such as students, education, skills, competencies, work experience, and employers, contained both main keywords—leadership and employability. Thus, the link between leadership and employability relates to the aforementioned topics, examining education and work-related topics. From the co-occurrence analysis, we argue that employability researchers focus on major topics such as employment, work, (higher) education, careers, skills, and competencies. From 1972 to 2021, there was an increase to the year-wise number of articles on employability and leadership. However, we are interested in the influence of supervisors' leadership as a job resource, and thus in a work context, on employees' employability. A systematic literature review is thus relevant to revealing the current state of research on the employability-leadership link from an employer-employee perspective, thus within work contexts. We are thus able to identify research gaps and develop an agenda for new research directions to further explore these topics from that perspective.

### 2.2. Main study: systematic literature review

#### 2.2.1. Methods

**Figure 5:**
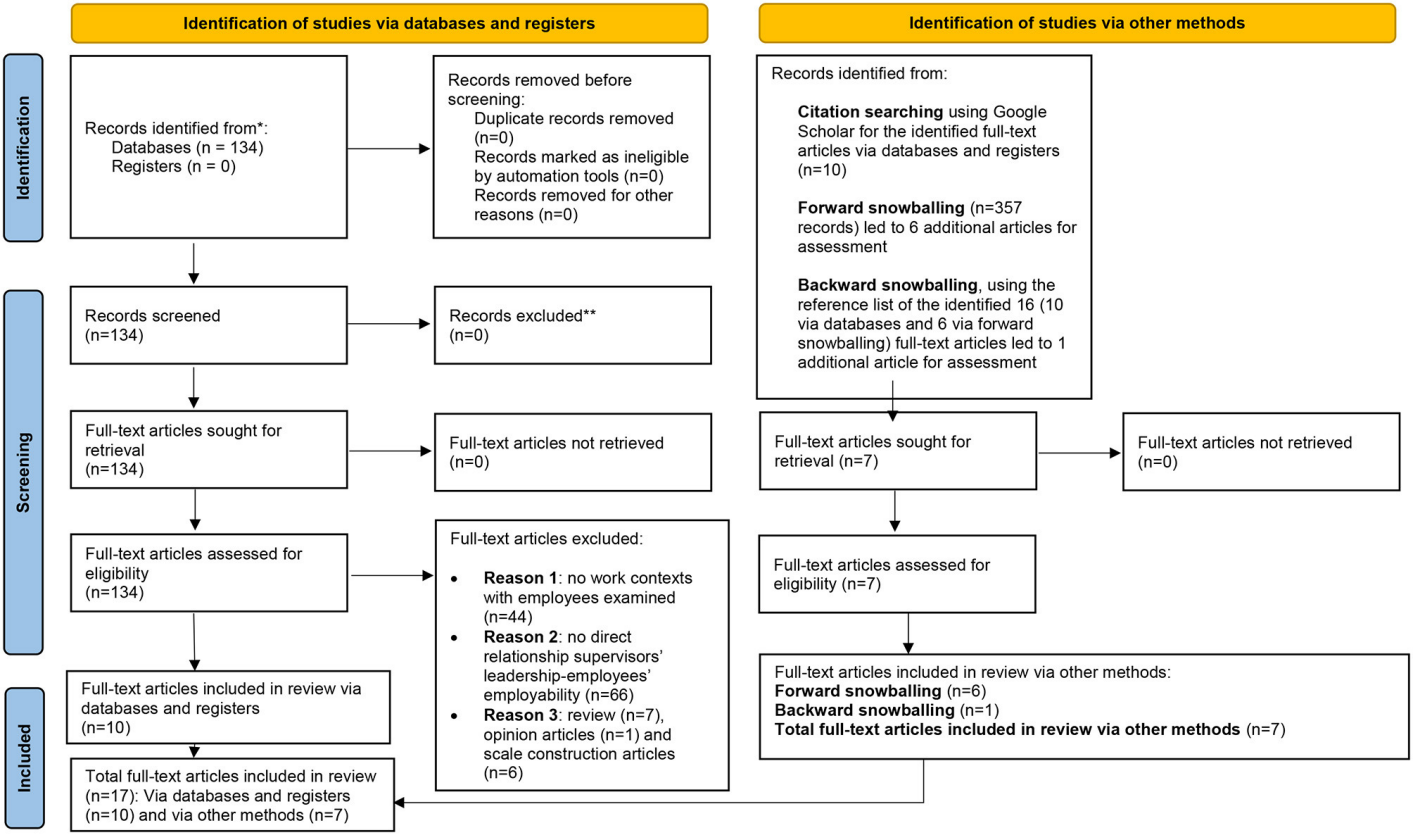
Flowchart of study selection using PRISMA 2020.

This literature review uses PRISMA 2020's recommendations regarding producing high-quality reporting (Moher et al., 2009; Page et al., 2021). Following Page et al. (2021)'s PRISMA 2020 flow diagram (Figure 5), this article reports on PRISMA's process to explore supervisors' leadership influence on employees' employability. To conduct the review, we use Grant and Booth (2009)'s critical four stages—Search, AppraisaL, Synthesis, and Analysis (SALSA)—so that the four principles of systematic reviews—transparency, inclusivity, explanatory, and heuristic nature—are guaranteed (Denyer and Tranfield, 2009). Transparency means being clear and explicit about methodologies, procedures, and processes, such that readers are able to audit them. Inclusivity requires assessing only studies that meet inclusion criteria, and subsequently addressing a research question. Explanatory means synthesizing findings from studies so that the findings, in combination, “make a whole that should be more than the sum of the parts” (680). Heuristic means reporting findings in a way that they have practical implications for practitioners and policymakers (Denyer and Tranfield, 2009).

Drawing from SALSA (Grant and Booth, 2009) and guided by the review's purpose, the Search stage identified relevant literature using multiple search techniques, such as database searching using free-text searches, with limited function and reference list checking (e.g., backward and forward snowballing). The AppraisaL stage comprised selecting studies systematically by assessing whether each met the inclusion criteria. Synthesis is concerned with seeking patterns, for example, by comparing findings from the reviews' included studies (Gray and Malins, 2004). The Analysis stage reports on the robustness of the Synthesis in terms of the quality of included studies (Denyer and Tranfield, 2009) that reasonably demonstrate the current state of research regarding what is and is not known (Denyer and Tranfield, 2009) about the influence of supervisors' leadership on employees' employability.

From January 2022 to 8 February 2022, we developed a research protocol that began with determining initial search terms, and inclusion and exclusion criteria, and with searching research databases, such as Academic Search Elite (EBSCO), APA PsycARTICLES (EBSCO), Emerald Insight (management) Psychology, Behavioral Sciences Collection, and WoS, to identify published peer-reviewed English and Dutch empirical articles. We did not exclude conference articles, books, and book chapters. The initial search term was based on core concepts of the aforementioned research question—leadership and employability—and was used to identify as many articles as possible, up to February 2022. Employability has been studied from many perspectives, which led to a plurality of definitions and operationalizations of the concept. The current review uses HRM and career perspectives that encompass focal stakeholders in organizations—employees and employers (Fugate et al., 2021).

#### 2.2.2. Eligibility criteria

We used inclusion and exclusion criteria to select studies that investigated the influence of leadership on employability among employees (i.e., subordinates) and supervisors (i.e., leaders). We searched major research databases using advanced filters, such as subjects that included “employability” OR “perceived employability” AND “leadership.” Searches were restricted to English- and Dutch-language peer-reviewed articles because such articles identify validated knowledge (Podsakoff et al., 2005), and there is lack of knowledge or resources for clear translations. Table 1 reports our inclusion and exclusion criteria.

**Table 1:** Inclusion and exclusion criteria.

**Inclusion criteria**	**Motivation**
Empirical studies on the influence of supervisors' leadership (e.g., managers, mentors, and coaches on employees' employability.	
Peer-reviewed journals	Explicitly identify validated knowledge (Podsakoff et al., 2005).
Content types	Published empirical articles in peer-reviewed journals, conference articles, books, and book chapters
Type of data	Quantitative and qualitative data
Subjects	Employability, perceived employability, and leadership
Contexts and participants	Employees and supervisors in work contexts
Publication date	No restriction
Language	English or Dutch
**Exclusion criteria**	**Exclusion criteria Motivation**
Language: non-English or Dutch	Lack of resources for clear translation
Content types	Repetitive articles such as reviews and opinion articles. Scale construction articles

From January 2022 to 8 February 2022, the first and second authors independently initially searched multiple major research databases using a combination of search techniques, such as Boolean searching. The Appendix reports the full list of research databases that we searched. From a preliminary search, we identified “self rated employability” and “competence-based employability” as additional keywords, resulting in a final search string that contained the keywords “*perceived employability”* OR “*self rated employability”* OR “*self rated employability”* OR “*competence-based employability”* OR “*competence based employability”* AND “*leadership.”* For inclusion, and using free-text searching with a limit function, the search string was restricted to language “*English”* OR “*Dutch”* AND “*peer-reviewed publications,”* with additionally content type “*all,”* publication date “*all,”* subjects “*employability,” “perceived employability,”* and “*leadership,”* which resulted in 134 articles that were included during full-text analysis.

#### 2.2.3. Results

##### 2.2.3.1. Study selection

Of the 134 articles found during the searches, 10 met the inclusion criteria. During the last stage of appraisal, the first and second authors independently conducted the forward and backward snowballing technique (Wohlin, 2014) to discover additional articles based on the 10 already identified. Forward snowballing discovers additional articles forward in time (Wohlin, 2014). We used Google Scholar to examine authors cited in the 10 articles. Forward snowballing revealed 357 additional articles, which the first and second authors reviewed independently by assessing the articles' titles and abstracts. We included articles that met the inclusion criteria reported in Table 1. Forward snowballing returned 6 additional articles. The first and second authors also independently used backward snowballing (Wohlin, 2014) to identify additional articles based on the 16 found during the initial searches and using forward snowballing. Backward snowballing discovered additional articles that met the inclusion criteria backward in time by using the reference lists in the 16 articles (Wohlin, 2014). The first and second authors examined the titles in the reference lists of the 16 articles, which resulted in 1 additional article that met the inclusion criteria. The researchers agreed on using these 17 articles as the final set to be analyzed (for a PRISMA flowchart, see Figure 5).

##### 2.2.3.2. Study characteristics

**Figure 6:**
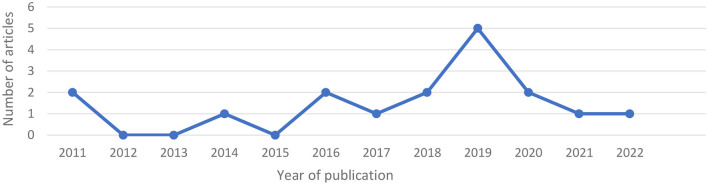
Yearly publication of included articles on the relationship between leadership and employability.

Since 2011, researchers have examined the influence of supervisors' leadership on employees' employability, and since 2016, they have published at least one empirical, peer-reviewed study each year (Figure 6).

Regarding context, and work contexts particularly, researchers have studied the relationship between leadership and employability in both non-profit and for-profit organizations. Most of the studies assessed in the current study (5), were conducted in education contexts, such as universities (Camps and Rodríguez, 2011; Van der Heijden and Spurk, 2019), higher vocational education institutions (Bhattacharya and Neelam, 2018; Gustari and Widodo, 2020), and primary schools (Struzyna and Marzec, 2017), followed closely by 3 studies conducted in small- and medium-sized enterprises (SMEs) (Bozionelos et al., 2016; Stoffers et al., 2019; Epitropaki et al., 2021). Building (Van der Heijden and Bakker, 2011; Van der Heijde and Van der Heijden, 2014), and healthcare organizations, such as non-academic hospitals (Van Harten et al., 2016), acute-care hospitals (Matsuo, 2022), and public healthcare units (Struzyna and Marzec, 2017), have been assessed in several studies. Multiple public service (Struzyna and Marzec, 2017), information and communication technology (Wang et al., 2019), food and beverage (Chughtai, 2019), automotive (Böttcher et al., 2018), insurance (Park, 2020), and mixed-industry (e.g., construction, manufacturing, finance, insurance, and communication; Yizhong et al., 2019) organizations have been assessed.

Regarding addressing participation by employees and supervisors, in most of the studies (8), data were collected in European organizations (Bass and Avolio, 1990a; Van der Heijden and Bakker, 2011; Van der Heijde and Van der Heijden, 2014; Bozionelos et al., 2016; Van Harten et al., 2016; Böttcher et al., 2018; Stoffers et al., 2019; Van der Heijden and Spurk, 2019), followed closely by Asia in 6 studies (Chughtai, 2019; Wang et al., 2019; Yizhong et al., 2019; Gustari and Widodo, 2020; Park, 2020; Matsuo, 2022) and South America (Camps and Rodríguez, 2011) (see Table 1). Two studies did not report in which countries they were conducted (Struzyna and Marzec, 2017; Bhattacharya and Neelam, 2018).

Regarding methodology, and excepting one study, all studies used quantitative survey designs. Bhattacharya and Neelam (2018) used a mixed-methods design to conduct both qualitative and quantitative research (Saunders et al., 2012). Table 2 reports details on participants, conceptualizations of the constructs under study, and other information.

**Table 2:** Overview of countries in which data were collected.

**Country**	**Number of articles**
The Netherlands	6
China, Germany, Greece, Italy, and Poland	2
Belgium, Costa Rica, Great Britain, Indonesia, Japan, Norway, Pakistan, and South Korea	1
Unspecified	2

Some studies collected data in multiple countries, and thus the total number of articles in combination with the countries in which the studies were conducted exceeded the 17 included articles.

##### 2.2.3.3. Measurements dependent variable: employees' perceived employability

Excepting Bhattacharya and Neelam (2018)'s qualitative measure of employees' employability, all studies used a quantitative design, measuring employability quantitatively as a dependent variable. Employability is operationalized disparately across the quantitative studies using validated scales, in which employees reported their perceived employability on questionnaires.

Seven studies (Camps and Rodríguez, 2011; Van der Heijden and Bakker, 2011; Van der Heijde and Van der Heijden, 2014; Bozionelos et al., 2016; Stoffers et al., 2019; Van der Heijden and Spurk, 2019; Park, 2020) assessed employability using Van der Heijde and Van der Heijden (2006)'s 47-item instrument, which consists of five dimensions—occupational expertise (15 items), anticipation and optimization (i.e., whether an individual responds to changes to internal and external job markets; 8 items), personal flexibility (8 items), corporate sense (7 items), and balance (i.e., balancing personal and a team's or organization's preferences; 9 items). Two studies (Park, 2020; Epitropaki et al., 2021) used a shortened version of Van der Heijde and Van der Heijden (2006)'s 5-factor employability instrument, a 22-item, short-form instrument that also measures the five dimensions—occupational expertise (5 items), anticipation and optimization (4 items), personal flexibility (5 items), corporate sense (4 items), and balance (4 items). Two studies (Struzyna and Marzec, 2017; Gustari and Widodo, 2020) used (De Lange et al., 2021)'s 47-item, 5-factor employability instrument to construct a valid fit-for-purpose employability scale, tailored to those studies. Drawing from Van der Heijde and Van der Heijden (2006), Struzyna and Marzec (2017) constructed a fit-for-purpose, 46-item employability scale that comprises eight dimensions of public-organization employees' employability—social competences, adjustability to changes, civic competences, knowledge and professional skills, ability to maintain balance, ethical competences, professional proactivity, and anticipatory striving for professional development. Gustari and Widodo (2020) use a self-constructed fit-for-purpose, 10-item employability scale based on Van der Heijde and Van der Heijden (2006)'s 47-item scale, which also comprises 5 dimensions, though labeled differently—specific work skills and competencies that are more general, proactivity, adaptability, work feelings, and balance. Böttcher et al. (2018) and Yizhong et al. (2019) use Rothwell and Arnold (2007)'s 11-item perceived employability scale, which assesses two dimensions—internal (4 items) and external employability (7 items). The dimensions associate with employees' ability to remain employable in (e.g., internal labor market) and outside (e.g., external labor market) the organization (Rothwell and Arnold, 2007). Van Harten et al. (2016) assessed employees perceived employability using two constructs—up-to-date expertise and willingness to change. Up-to-date expertise was measured using Thijssen and Walter (2006)'s 9-item, 3-dimension scale, which contains the dimensions technical expertise (3 items), economic expertise (3 items), and perceptional expertise (3 items). Willingness to change was measured using a self-constructed fit-for-purpose, 4-item scale based on Wittekind et al. (2010) and Van Dam (2004). Multiple studies treated perceived employability as a unidimensional construct. Chughtai (2019) used De Vos and Soens (2008) 3-item scale, Wang et al. (2019) used Eby et al. (2003)'s 6-item scale, and Matsuo (2022) used De Cuyper et al. (2011)'s 4-item scale. Table 3 reports greater details on these studies' participants, conceptualizations of constructs, and other information.

**Table 3:** Details of included studies.

**References**	**Empirical context**	**Research design**	**Sample/participants**	**Conceptualization of leadership**	**Conceptualization of employability**	**Theories used in research frameworks**
Van der Heijden and Bakker (2011)	• The Netherlands • One large Dutch building company	• Quantitative cross-sectional survey research • Mediation research model	Employees/supervisors pairs (*n* = 303)	• Perceived supervisors' transformational leadership assessed using employee ratings • Five of the nine original subscales (45 items) from Alimo-Metcalfe and Alban-Metcalfe (2001)'s transformational leadership questionnaire; α ranged from 0.82 to 0.95	• Perceived employees' employability measured using supervisor ratings for a maximum of three employees • Van der Heijde and Van der Heijden (2006)'s 47-item, 5-factor employability instrument; α ranged from 0.83 to 0.95 within dimensions	Job demands-resources (JD-R) theory (Bakker and Demerouti, 2017)
Camps and Rodríguez (2011)	• Costa Rica • One large university	• Quantitative cross-sectional survey research • Two-level data structure • Department-level (*n* = 75) • Employee-level • Moderated mediation model	Employees (*n* = 795)	• Perceived Head of Department leader's transformational leadership assessed using lecturers' and professors' ratings • Perceived transformational leadership measured using Podsakoff et al. (1990)'s 5-dimensional scale (18 items)	Perceived employees' employability measured using Van der Heijde and Van der Heijden (2006)'s 47-item, 5-factor employability instrument	Social exchange theory (SET; Cropanzano and Mitchell, 2005; Blau, 2017)
Van der Heijde and Van der Heijden (2014)	The Netherlands One large Dutch building company	• Quantitative cross-sectional survey research • Main effect research model • Multi-source data collection	• Employees (*n* = 314) • Immediate supervisors (*n* = 334) • Employee/supervisor pairs (*n* = 290)	• Perceived supervisors' transformational leadership assessed using employee ratings • Five of the nine original subscales (45 items) of Alimo-Metcalfe and Alban-Metcalfe (2001)'s transformational leadership questionnaire; α ranged from 0.82 to 0.95	• Perceived employees' employability assessed using both self-ratings and supervisor ratings of Van der Heijde and Van der Heijden (2006)'s 47-item, five-factor employability instrument; α ranged from 0.78 to 0.90 for self-ratings, and 0.83 to 0.95 for supervisor ratings	Authors revealed no theory for explaining the findings.
Van Harten et al. (2016)	• The Netherlands • Three Dutch non-academic hospitals	• Quantitative cross-sectional survey research • Mediated research model	• Employees (*n* = 1,626): • Nursing staff (39%), medical office assistants or clerical staff (25%), non-nursing medical employees (24%), middle or higher managers or staff members (12%).	• Perceived supervisors' support of employees' wellbeing and functioning assessed using Knies and Leisink (2014)'s 4-item scale; α was 0.91 • Perceived supervisors' support of employees' development assessed using Knies and Leisink (2014)'s 4-item scale; α was 0.87	• Perceived employees' employability measured using two constructs: • Thijssen and Walter (2006)'s 3-dimension, 9-item, up-to-date expertise scale; α was 0.78, and willingness to change assessed using a 4-item scale based on Wittekind et al. (2010) and Van Dam (2004); α was 0.71	Authors revealed no theory for explaining the findings.
Bozionelos et al. (2016)	• Greece, Italy, and Poland • Small and medium-sized enterprises (SMEs)	• Quantitative cross- sectional survey design • Moderated mediation model • Multi-source data collection: IT professionals and line managers	Information technology (IT) professionals (*n* = 207) from Greece (*n* = 50), Italy (*n* = 43), and Poland (*n* = 114)	Perceived mentoring receipt assessed using Dreher and Ash (1990)'s 5-item scale; α was 0.83	Perceived employees' employability assessed using supervisor ratings of Van der Heijde and Van der Heijden (2006)'s 47-item, 5-factor employability instrument; α ranged from 0.81 to 0.92 within dimensions	Authors revealed no theory for explaining the findings.
Struzyna and Marzec (2017)	• Unspecified • Several public organizations: • Municipal/commune centers of culture (*n* = 14) • Public health care units (*n* = 14) • County labor offices (*n* = 14) • Municipal/commune units of social assistance (*n* = 14) • Primary schools (*n* = 14) • Commune centers for family support (*n* = 44) • Municipal commune offices (*n* = 33)	• Quantitative cross- sectional survey design • Mediated model	• Employees (*n* = 566) specified by public organizations: • Municipal/commune centers of culture (*n* = 80) • Public health care units (*n* = 80) • County labor offices (*n* = 80) • Municipal/commune units of social assistance (*n* = 80) • Primary schools (*n* = 80) • Commune centers for family support (*n* = 80) • Municipal commune offices (*n* = 86)	• Perceived quality of supervisor-employee relationships (i.e., LMX) measured using Liden and Maslyn (1998)'s scale • Perceived transformational leadership was measured with a constructed scale based on scales developed by Hartog et al. (1997) and Avolio et al. (1999).	• Perceived employees' employability measured using an 8-dimensional constructed scale, drawing from Van der Heijde and Van der Heijden (2006)'s 47-item, 5-factor employability instrument • The scale contains the dimensions of social competences, ability to adjust to changes, civic competences, knowledge and professional skill, ability to maintain balance, ethical competences, pro-active professional attitude, anticipatory strive for professional development	LMX (Graen and Uhl-Bien, 1995)
Böttcher et al. (2018)	• Germany • Two organizational units in one large German automotive company	• Quantitative cross- sectional survey design • Moderated model	White-collar employees (*n* = 1,006)	• Perceived transformational leadership assessed using a 20-item, multi-dimensional German version of the Multifactor Leadership Scale 5X short (Felfe, 2006), developed by Bass (1985) • This multi-dimensional scale contains five dimensions (each dimension assesses 4 items), (I) idealized influence attribute, (II) idealized influence behavior, (III) inspirational motivation, (IV) intellectual stimulation, and (V) individual consideration; α ranged from 0.80 to 0.91, and 0.96 for the overall scale	• Perceived employability measured using Rothwell and Arnold (2007)'s 11-item scale, which contains sub-dimensions internal employability (four items; α = 0.55) and external employability (seven items; α = 0.79)	Authors revealed no theory for explaining the findings.
Bhattacharya and Neelam (2018)	Unspecified One business school Two large multinational conglomerates, two retail companies, two financial services companies and three IT companies	• Mixed-method research design with two data collections: first was a quantitative survey and second was semi-structured, in-depth interviews • Multi-source data collection	• Quantitative survey: first year students/interns (*n* = 110) from human resources, sales/marketing, operations, and finance • Qualitative research using semi-structured, in-depth interviews among 14 pairs of students/interns and their mentors from two large multinational conglomerates (*n* = 4), two retail companies (*n* = 2), two financial services companies (*n* = 2), and three IT companies (*n* = 6)	Characteristics of supervisors that leads to intern's satisfaction from LMX-perspective.	Intern's satisfaction in terms of future employability	LMX (Graen and Uhl-Bien, 1995)
Yizhong et al. (2019)	• China • Organizations in industries such as construction, manufacturing, finance, insurance, and communications	• Quantitative survey with two waves of data collection • Mediated model	Employees (*n* = 760) who worked under line manager for more than 1 year	• Perceived transformational leadership assessed using the 20-item, 5-dimensional Multifactor Leadership Scale (Bass and Avolio, 1995). • This 5-dimension scale contains dimensions (I) idealized influence attribute, (II) idealized influence behavior, (III) inspirational motivation, (IV), intellectual stimulation, and (V) individual consideration; α was 0.938 for the overall scale	• Perceived employability assessed using Rothwell and Arnold (2007)'s 10-item scale, which contains sub-dimensions internal and external employability; α was 0.882 for the overall scale	• Job characteristics theory (Hackman and Oldham, 1975) • Social exchange theory (SET; Cropanzano and Mitchell, 2005; Blau, 2017) • LMX (Graen and Uhl-Bien, 1995)
Chughtai (2019)	• Pakistan • One large food and beverage company	• Quantitative cross- sectional survey design • Mediated model	Full-time employees (*n* = 176) who worked in the head office	Perceived servant leadership measured using Liden et al. (2015)'s 7-item servant leadership scale; α was 0.86	Perceived employability measured using De Vos and Soens (2008)'s 3-item scale; α was 0.84	Authors revealed no theory for explaining the findings.
Van der Heijden and Spurk (2019)	• The Netherlands • One University	• Quantitative cross- sectional survey design • Moderated model	Academic employees across science, technology, engineering and mathematics, and social science disciplines (*n* = 139)	Perceived LMX measured using Graen and Uhl-Bien (1995)'s 7-item scale; α was 0.92	Perceived employability measured using Van der Heijde and Van der Heijden (2006)'s 47-item, 5-factor employability instrument; α ranged from 0.79 to 0.92 within dimensions	• Job demands-resources (JD-R) theory (Bakker and Demerouti, 2017) • Conservation of resources (COR) theory from Hobfoll (1989) • LMX (Graen and Uhl-Bien, 1995)
Wang et al. (2019)	• China • One internet technology company	• Quantitative survey design with two waves of data collection • Moderated mediation model	Employees (*n* = 283)	Perceived servant leadership measured using Liden et al. (2015)'s 7-item servant leadership scale; α was 0.89	Perceived employability measured using Eby et al. (2003)'s 6-item scale; α was 0.89	Authors revealed no theory for explaining the findings.
Stoffers et al. (2019)	• Belgium and the Netherlands • Small and medium-sized enterprises (SMEs)	• Quantitative cross- sectional survey design • Moderated mediation model	• Employees/immediate supervisor pairs: • Belgian sample (*n* = 105) • Dutch sample (*n* =4 87)	Perceived LMX measured using Graen and Uhl-Bien (1995)'s 7-item, multi-dimensional scale, which contains dimensions respect (two items, α 0.70 to 0.76), trust (two items, α 0.61 to 0.69), obligation (two items, α 0.74 to 0.75), and relationship quality (one item)	Perceived employability measured using Van der Heijde and Van der Heijden (2006)'s 47-item, 5-factor employability instrument; α ranged in the Belgian sample from 0.75 to 0.90, and in the Dutch sample from 0.78 to 0.91	LMX (Graen and Uhl-Bien, 1995)
Park (2020)	• South Korea • One life insurance company	• Quantitative cross- sectional survey design • Main-effects model	Employees (*n* = 257)	Perceived LMX measured using Graen and Uhl-Bien (1995)'s 7-item scale; α was 0.768	Perceived employability measured using Van der Heijden et al. (2018)'s 22-item, short-form, 5-factor employability instrument; α ranged from 0.790 to 0.892 within dimensions	LMX (Graen and Uhl-Bien, 1995)
Gustari and Widodo (2020)	• Indonesia • Three private higher-education institutions	• Quantitative cross-sectional survey design • Mediated model	Permanent (*n* = 170).	Perceived transformational leadership assessed using a modified, multi-dimensional, 12-item scale based on Bass and Avolio (1990b); α was 0.966	Perceived employability measured using a 10-item scale based on Van der Heijde and Van der Heijden (2006)'s 5-dimension scale; α was 0.875	No information about used theory to embed the findings within theory
Epitropaki et al. (2021)	• Germany, Greece, Italy, Norway, Poland, The Netherlands, and the United Kingdom • Small and medium-sized enterprises (SMEs)	• Quantitative cross- sectional survey design • Moderated mediation model	Information and communication technology (ICT) employees (*n* = 1,127) and 988 supervisors (*n* = 988); leader-follower dyads (*n* = 967)	Follower- and leader-rated perceived LMX measured using an adapted 7-item version of LMX-7 (Scandura and Graen, 1984; Graen and Uhl-Bien, 1995); α was 0.88 for follower-rated LMX and 0.85 for leader-rated LMX	Follower/employee-rated and leader-rated perceived employability measured using both leaders and followers from Van der Heijden et al. (2018)'s 22-item, short-form, 5-factor employability instrument; composite α was 0.89 for follower-rated employability and 0.94 for leader-rated employability	Sponsorship theory (Rosenbaum, 1979; Wayne et al., 1999)
Matsuo (2022)	• Japan • One acute care hospital	• Quantitative survey design with two waves of data collection • Mediated model	Nurses and assistant nurses (*n* = 221)	Perceived supervisor support for strengths use (PSSSU) was assessed using Keenan and Mostert (2013)'s 8-item scale; α was 0.97	Perceived employability measured using De Cuyper et al. (2011)'s 4-item scale; α was 0.84	• Job demands-resources (JD-R) theory (Bakker and Demerouti, 2017) • Positive psychology (Seligman, 2019)

##### 2.2.3.4. Measurement leadership as a determinant of employability enhancement: content and effects

**Figure 7:**
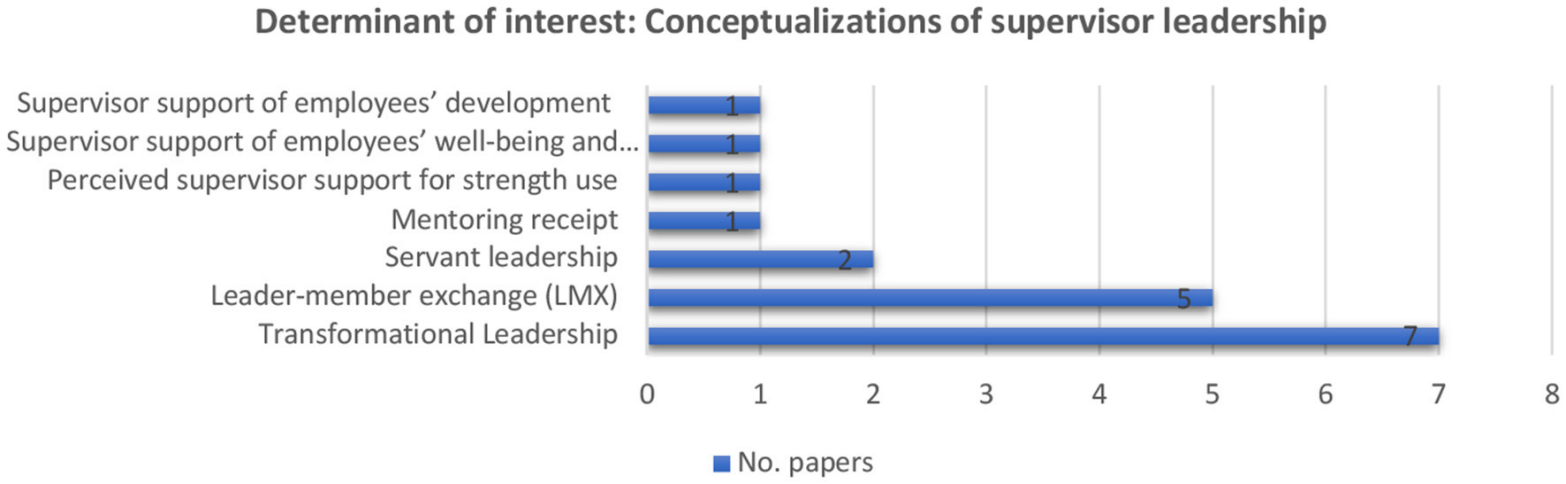
Conceptualizations of supervisor leadership in included articles (*n* = 17).

The determinant of interest is supervisors' leadership. We now discuss how such leadership was measured and contributes to employees' employability (See Figure 7 for conceptualizations of supervisor leadership in included articles). The 17 studies were heterogeneous concerning research models. The majority (Van der Heijden and Bakker, 2011; Van Harten et al., 2016; Struzyna and Marzec, 2017; Chughtai, 2019; Yizhong et al., 2019; Gustari and Widodo, 2020; Matsuo, 2022) examine leadership as a determinant of employability enhancement using a mediated model. Researchers also investigate the relationship between leadership and employees' employability using moderated (Böttcher et al., 2018; Van der Heijden and Spurk, 2019), moderated mediation (Camps and Rodríguez, 2011; Bozionelos et al., 2016; Stoffers et al., 2019; Wang et al., 2019; Epitropaki et al., 2021), mixed methods (Bhattacharya and Neelam, 2018), and main effects (Van der Heijde and Van der Heijden, 2014; Park, 2020) models.

Findings suggest that supervisors' leadership has been conceptualized in several ways. Most of the studies focus on relationships between transformational leadership (Burns, 1978) and employability. Transformational leadership contains four dimensions, the four I's (Avolio et al., 1991), including idealized influence (e.g., charisma), inspirational motivation, intellectual stimulation, and individualized consideration. These four I's encourage followers to transform their attitudes, values, and behaviors through empowerment, such that followers achieve outstanding performance (Burns, 1978; Bass and Avolio, 1990a; Bass, 1999). Idealized influence associates with a leader's behaviors that make employees perceive their leaders as role models. Inspirational motivation is concerned with leaders having a vision of a future state that inspires and motivates followers so that they achieve outstanding performance and increase job satisfaction. Intellectual stimulation describes a leader's behaviors that stimulate and recognize followers' creativity and innovation by, for example, fostering autonomy. Individualized consideration is concerned with a leader's behaviors that focus on followers' developmental needs through, for example, support and coaching (Bass and Avolio, 1990a; Bass, 1999).

Several authors have found that transformational leadership relates positively with employability (Van der Heijden and Bakker, 2011, ß = 0.14, *p* < 0.05; Camps and Rodríguez, 2011, ß = 0.62, *p* < 0.01; Van der Heijde and Van der Heijden, 2014, ß = 0.23, *p* < 0.001 for supervisor' ratings of employees' employability and ß = 0.17, *p* < 0.01 for employee's ratings of employability; Struzyna and Marzec, 2017, γ = 0.12, *p* < 0.01; Yizhong et al., 2019, ß = 0.21, *p* < 0.01; Gustari and Widodo, 2020, ß = 0.34, *p* < 0.01).

Böttcher et al. (2018) argue that the relationship between transformational leadership and internal employability (ß = 0.31, *p* < 0.01 for the full item scale and ß = 0.24, *p* < 0.01 for the one-item scale) is stronger than that with external employability (ß = 0.15, *p* < 0.01). They found that when the subdimensions of transformational leadership were entered simultaneously, three dimensions—idealized influence attributed (ß = 0.12, *p* < 0.05), idealized influence behavior (ß = 0.11, *p* < 0.05), and individualized consideration (ß = 0.20, *p* < 0.01)— had a greater positive influence on internal employability (full item scale), in contrast with inspirational motivation (ß = −0.45, *p* = 0.45, n.s.) and intellectual stimulation (ß = −0.05, *p* = 0.38, n.s.). When the subdimensions of transformational leadership were entered simultaneously, idealized influence attributed (ß = 0.15, *p* < 0.05) and individualized consideration (ß = 0.20, *p* < 0.01) also influenced internal employability (one-item scale) positively. This contrasts with non-significant influences of idealized influence behavior (ß = 0.02, *p* = 0.73, n.s.), inspirational motivation (ß = −0.00, *p* = 0.94, n.s.), and intellectual stimulation (ß = −0.05, *p* = 0.73, n.s.) on internal employability (one-item scale) when the subdimensions were entered simultaneously. When the subdimensions of transformational leadership were entered simultaneously, Böttcher et al. (2018) found no relationship between them and external employability. When entered separately, the idealized influence attributed (ß = 0.15, *p* < 0.01), idealized influence behavior (ß = 0.14, *p* < 0.01), inspirational motivation (ß = 0.13, *p* < 0.01), intellectual stimulation (ß = 0.11, *p* < 0.01), and individualized consideration (ß = 0.15, *p* < 0.01) influenced external employability positively.

Assessing the second most common leadership model, multiple authors report a positive influence of leader-member exchange (LMX) (Graen and Uhl-Bien, 1995) on employability. LMX represents perceived quality of social exchanges between followers and immediate supervisors, characterized by dyadic affection, loyalty, contribution, and professional respect (Liden and Maslyn, 1998), and their influence on both leaders' and followers' attitudes and behaviors (Graen and Uhl-Bien, 1995), such as employability.

Epitropaki et al. (2021) found that both follower- (ß = 0.570, *p* < 0.01) and leader-rated (ß = 0.632, *p* < 0.01) employability are influenced positively by agreement on LMX among followers and leaders. In the context of LMX disagreement, leader-rated employability was higher when leaders perceived that LMX was higher than followers' perceived LMX. Stoffers et al. (2019) find a positive influence of immediate supervisors' LMX on employees' employability, which was influenced by national context in Belgium and the Netherlands. Findings suggest that a positive relationship in a Dutch sample (ß = 0.341, *p* < 0.001) was greater than in a Belgium sample (ß = 0.296, *p* < 0.05). Struzyna and Marzec (2017) found that LMX fosters employees' employability (γ = 0.45, *p* < 0.01), but Van der Heijden and Spurk (2019) found no support for an influence of LMX on all dimensions of perceived employability (Van der Heijde and Van der Heijden, 2006)—occupational expertise, anticipation and optimization, personal flexibility, corporate sense, and balance. Park (2020) found that LMX associates with only one dimension of perceived employability (Van der Heijden et al., 2018)—corporate sense (ß = 0.245, *p* < 0.01).

Examining a third leadership model, Chughtai (2019) and Wang et al. (2019) demonstrate contrary findings of a positive influence of servant leadership (Greenleaf, 1977) on employees' perceived employability. Servant leadership concerns fulfilling followers' needs by providing support that subsequently enhances followers' potential (Greenleaf, 1977), such as career potential (e.g., employability). Wang et al. (2019) found that servant leadership fosters employees' perceived employability (ß = 0.19, *p* < 0.01), but Chughtai (2019) found no support for the same (ß = −0.09, *ns*).

Some studies instead assess employees' perceived supervisor support, with one suggesting that leadership influences employees' perceived employability positively, including informal mentoring (Bozionelos et al., 2016, ß = 0.26, *p* < 0.01), but perceived supervisor support does not (Matsuo, 2022, ß = −0.11, *ns*). Mentoring represents a relationship between two individuals, traditionally of unequal status, during which the mentor provides multiple development opportunities through, for example, coaching and counseling (Kram and Isabella, 1985). Supervisor support associates with supervisory support, which contributes to positive work outcomes, such as job satisfaction (Miglianico et al., 2020). Van Harten et al. (2016) demonstrate that supervisors' support of employees' wellbeing and functioning (ß = 0.24, *p* < 0.001) influences employability, conceptualized as up-to-date expertise, positively, but not employability conceptualized as willingness to change. Supervisors' support of employees' development influences employability conceptualized as willingness to change, but not that conceptualized as up-to-date expertise. Table 3 reports greater details on these articles.

## 3. The mechanisms: mediators and moderators

While reporting on direct influences of supervisor leadership on employee employability, findings from the 17 articles suggested that the relationship between leadership and employability can be explained by several mechanisms, such as mediators, moderators, or both. Mechanisms represent “the basis for the effect, i.e., the processes or events that are responsible for the change; the reasons why change occurred or how change came about” Kazdin (2007, p. 3). Van der Heijden and Bakker (2011) found that work-related flow is a mediator and thus represents a mechanism in the positive relationship between transformational leadership and employability. Work-related flow is “a short-term peak experience at work that is characterized by absorption, work enjoyment and intrinsic work motivation” (Bakker, 2005, p. 27).

Van der Heijde and Van der Heijden (2014) demonstrate that the influence of leadership on employability enhancement is moderated by both an employee's work role (e.g., managerial/no managerial work role) and personality. Findings suggest that among employees without a managerial work role, the relationship between transformational leadership and employability was positive only for supervisors' ratings of employability (ß = 0.35, *p* < 0.001) and not for employees' ratings of the same. However, among employees who had a managerial work role, the relationship was positive for both supervisors' (ß = 0.17, *p* < 0.05) and employees' (ß = 0.22, *p* < 0.01) ratings (Van der Heijde and Van der Heijden, 2014).

Böttcher et al. (2018) argue that transformational leadership, particularly idealized influence behavior and individual consideration, moderates the negative relationship between age and both internal and external employability. Transformational leadership diminishes the negative influence of age on internal employability, contrary to external employability. Yizhong et al. (2019) found that job characteristics, such as job demands, skill discretion, and decision authority, and social exchanges, particularly perceived organizational support and team member exchanges, explain the positive effects of leadership on employability. Chughtai (2019) demonstrate that servant leadership indirectly and positively influences employability through mediators such as proactive career behaviors (e.g., career planning, skill development, and networking).

Van der Heijden and Spurk (2019) use leadership, particularly LMX, to explain the relationship between learning value of a job and employability. In a context of LMX, relationships between learning value of a job and all five dimensions of employability (Van der Heijde and Van der Heijden, 2006) are positive and strengthened. Wang et al. (2019) demonstrate a positive association between servant leadership and employability, influenced by two mechanisms—career skills (i.e., a mediator) and proactive personality (i.e., a moderator). Servant leaders foster employees' career skills, which subsequently enhance employees' employability. The degree of a proactive personality strengthens the influence of servant leadership on career skills, which subsequently enhances employees' employability. Stoffers et al. (2019) demonstrate that employees' national context (i.e., Belgium and the Netherlands) moderates minimally the positive influence of LMX on employability. Matsuo (2022) found that perceived supervisor support enhances employees' employability (De Cuyper and De Witte, 2011) through the mechanism (i.e., a mediator) strength.

## 4. Discussion

This review is first to provide an overview of the influence of supervisors' leadership on employees' employability at work (i.e., internal, external, and subdimensions of employability). Despite a lack of studies that assess leadership as a predictor of employability enhancement, we identify and review 17 empirical articles, most of which report positive relationships. This review suggests that supervisors' leadership influences employees' employability positively, thus demonstrating that investment in such leadership represents a valuable HRM strategy regarding employability.

As a result of the preliminary study, and according to Chughtai (2019) and Wang et al. (2019), we argue that there exists scant research that assesses the leadership-employability link explicitly. To develop a research agenda from an employer-employee relationship perspective, it is paramount to recognize the current state of research on the leadership-employability relationship (Fugate et al., 2021). When talent is scarce and retaining employees is important to employers, identifying how leadership, as a job resource, contributes to employability enhancement is crucial.

To explain the positive influences, both direct and indirect, of supervisors' leadership on employees' employability, researchers use various theories of perceived support from leaders in terms of job resources, and subsequent outcomes such as employability. We found that the 17 reviewed articles use social exchange perspectives to explain this influence. From a social exchange perspective, the articles use social exchange theory (SET; Cropanzano and Mitchell, 2005; Blau, 2017) (*n* = 2) or LMX (Graen and Uhl-Bien, 1995; *n* = 6) to explain the positive influence of supervisors' leadership on employees' employability. SET and LMX suggest that two-way social exchange relationships between supervisors and employees affect employees' perceptions of organizational support strongly (Wayne et al., 1997). High LMX is dominated by high dyadic respect, trust, commitment, and interest in each other's wellbeing, which subsequently foster employees' access to job resources (e.g., support and both informal and formal learning opportunities). Employees' improved access to job resources positively affects workplace outcomes (Liden et al., 1997) such as employability.

The articles also use other theories to explain the positive influence of supervisor leadership on employee employability. Van der Heijden and Spurk (2019) and Matsuo (2022), for example, use job demands-resources (JD-R) theory (Bakker and Demerouti, 2017). According to Bakker and Demerouti (2017), JD-R involves a human resources management approach, suggesting that job resources, defined as “physical, psychological, social or organizational aspects of the job are functional in achieving work goals” (p. 274), influence employees' work motivation, especially under challenging work conditions. Job resources, such as performance feedback, LMX, and opportunities for growth, help employees cope with job demands, which subsequently enhance employability. Drawing on this theory, Matsuo (2022) found that a positive psychology (Seligman, 2019) lens explains the link between supervisors' leadership and employees' employability. As a job resource, supervisor support fosters employees' confidence to succeed with task performance, and the result such confidence is employees succeeding with their task performance, which enhances work performance (Van Woerkom et al., 2016) and influences employability positively.

To explain the positive influence of supervisor leadership on employee employability, Van der Heijden and Spurk (2019) use conservation of resources (COR) theory from Hobfoll (1989), which is generally similar to Yizhong et al. (2019)'s use of job characteristics theory (Hackman and Oldham, 1975). COR associates with JD-R theory (Bakker and Demerouti, 2017) because both focus on employees who use resources to cope with organizational stress such as job insecurity. According to Hobfoll et al. (2018), COR suggests that “individuals strive to obtain, retain, foster and protect those things they centrally value” (p. 104), such as employment. Using this coping strategy, employees obtain and retain resources by, for example, investing in skill development (i.e., a personal resource) and LMX, the quality of a supervisor-employee relationship (i.e., a social resource; Hobfoll et al., 2018). Froehlich et al. (2019) demonstrate that skill development through task variety fosters employability indirectly, and Wayne et al. (1997) argue that degree of access to job resources is affected by the quality of supervisor-employee relationships, which subsequently contributes to employees' employability enhancement.

Job characteristics (JC) theory (Hackman and Oldham, 1975) appears suitable to explaining the positive influence of supervisor leadership on employee employability. JC (Hackman and Oldham, 1975) suggests that resources such as autonomy, job feedback, task significance, and task identity influence feelings and behaviors, such as motivation. From this perspective, supervisor leadership, as a contextual resource, influences employees' work experiences (i.e., work meaningfulness and responsibility for work outcomes), which subsequently enhance employability.

According with JD-R, COR, and JC theory, Epitropaki et al. (2021) use sponsorship theory (Rosenbaum, 1979; Wayne et al., 1999), to explain the positive link between supervisors' leadership and employees' employability. The theory suggests that supervisors provide resources such as sponsoring activities and career mentoring, and they subsequently contribute to career success and thus employability. Sponsorship theory is, therefore, a suitable lens to explain the positive association between supervisor leadership and employee employability.

Excepting Bhattacharya and Neelam (2018)'s mixed-method study, a design that combines quantitative and qualitative methods (Creswell, 1999), all studies used cross-sectional, quantitative, and deductive (i.e., hypothesis testing) approaches. All also collected, at most, two waves of data, which means that they used cross-sectional, not longitudinal, designs. Ployhart and MacKenzie (2015) argue that longitudinal designs include three waves of measurement of the same variables, which provides insights into cause-and-effect (i.e., causality) among them. Since cross-sectional designs involve one-time specific measurement from a single source (e.g., a respondent), the design inherently includes validity concerns, such as common method bias and causal inference (Rindfleisch et al., 2008). We are thus unable to argue a case for causal relationships between supervisor leadership and employee employability (Setia, 2016).

Despite the advantages of quantitative, deductive hypothesis testing, including the ability to conduct surveys quickly, quantitative research does not include in-depth analyses of individuals' characteristics (e.g., beliefs, values, and assumptions; Neuman, 2014). Due to the quantitative designs used in the studies assessed, we are unable to discover greater insights into participants' behaviors, and thus we are able only to test existing theories, instead of constructing new ones (Neuman, 2014).

The studies were conducted in various work contexts, such as educational, SMEs, healthcare, and several industries at once, and the studies' contexts also varied geographically. Some were conducted in Europe (Van der Heijden and Bakker, 2011; Van der Heijde and Van der Heijden, 2014; Bozionelos et al., 2016; Van Harten et al., 2016; Böttcher et al., 2018; Stoffers et al., 2019; Van der Heijden and Spurk, 2019; Epitropaki et al., 2021), followed closely by Asia (Chughtai, 2019; Wang et al., 2019; Yizhong et al., 2019; Gustari and Widodo, 2020; Park, 2020; Matsuo, 2022) and North America (Camps and Rodríguez, 2011). This suggests that we found support for a mostly positive relationship between supervisor leadership and employee employability in various work contexts across countries. However, from a “cross-context scholarship” (Whetten, 2009, p. 29) perspective is this an important finding. Namely, outcomes of supervisors' leadership are influenced by context effects (Oc, 2018) defined as “the set of factors surrounding a phenomenon that exert some direct or indirect influence on it (Whetten, 2009, p. 31). Namely, overall, and based on the studies assessed, we argue that results do not differ greatly among these work contexts.

Research conceptualizes supervisor leadership and employee employability in several ways. We argue that leadership influences, that is enhances, at least one dimension of employees' employability both directly and indirectly. Chughtai (2019) found contradictory results; that is, no support for a direct influence of supervisors' leadership on employees' employability. We refer to Fiedler (1964)'s contingency theory of leadership as an explanation for different results within several conceptualizations of the constructs. Fiedler (1964) suggests no universal leadership style that fits every context.

Findings suggest that major leadership concepts, such as transformational leadership, LMX, and servant leadership, and leadership concepts related to employees' support of employees' development, perceived supervisor support of employees' wellbeing and functioning, and perceived supervisor support for strength use and mentoring, are leader-support leadership concepts (Cheong et al., 2019) that have a positive and direct influence on employability. Such leadership encourages followers to transform their attitudes, values, and behaviors through empowerment so that followers achieve outstanding performance (Burns, 1978; Bass, 1985, 1999). Transformational leaders motivate employees by being role models, sharing inspired visions of a desired future, and recognizing and stimulating employees' creativity and development (e.g., stimulate self-development) (Bass, 1999), and using power and authority to focus on change (e.g., progress and development; Tucker and Russell, 2004), which subsequently enhances employability. Similar to Bass (1999), Greenleaf (1977) also focuses on transforming employees' attitudes, values, and behaviors by putting service first instead of leading, such that employees “grow healthier, wiser, freer, more autonomous and more likely themselves to become servants” (Greenleaf, 1977, p. 13). LMX represents the quality of social exchanges between a leader and follower, which associate with exchanges of job resources and subsequently influence both leaders' and followers' behaviors (Wayne et al., 1997). The quality of dyadic relationships between leaders and followers determines the extent to which leaders offer valuable resources such as training and feedback (Liao et al., 2009), which subsequently enhance employees' employability (Froehlich et al., 2019). Perceived supervisor support of employee development, wellbeing, functioning, strength use, and mentoring associates with employees' perceptions of whether supervisors care about them and value their work (Eisenberger et al., 2002, p. 565). However, the strength of this positive influence differs among leadership conceptualizations. Transformational leadership (Camps and Rodríguez, 2011), followed closely by LMX (Epitropaki et al., 2021), are the types of leadership that associate most positively with employability. Wang et al. (2019) demonstrate a positive influence of servant leadership on employability, and in contradiction, Chughtai (2019) found no support for the same relationship. Instead of assessing explicit leadership styles, the remainder of the studies assess employees' perceived supervisor support, with inconclusive results reported. Depending on the conceptualization of employees' perceived supervisor support, such support might enhance employees' employability, but to a lesser degree than major leadership concepts such as transformational leadership and LMX.

Regarding the influence of supervisor leadership on employee employability, findings suggest the importance of mediators and moderators. Mediators such as work-related flow (Van der Heijden and Bakker, 2011), job demands, skill discretion, and decision authority (e.g., job characteristics), and social exchange mechanisms, particularly perceived organizational support and team-member exchange (Yizhong et al., 2019), career planning, skill development, and networking (e.g., proactive career behaviors; Chughtai, 2019), career skills (Wang et al., 2019), and strength use (Matsuo, 2022), are crucial to explaining the influence of supervisor leadership on employee employability. Moderators such as work roles, personality (Van der Heijde and Van der Heijden, 2014), proactive personalities (Wang et al., 2019), and national contexts (Stoffers et al., 2019) influence the relationship between supervisor leadership on employee employability. This implies that in addition to several leadership concepts and mediators discussed above, the positive influence of leadership on employability differs among work roles, personalities (Van der Heijde and Van der Heijden, 2014), proactive personalities (Wang et al., 2019), and national contexts (Stoffers et al., 2019). As mechanisms, both mediators and moderators are crucial to explaining the positive influence of supervisor leadership on employee employability.

### 4.1. Setting an agenda for employability research

This review suggests how supervisors' leadership influences employees' employability, but more research is needed to explore this relationship further. We therefore propose multiple directions for future research, setting an agenda, from (1) theoretical perspective that investigates how other, unapplied leadership frameworks and other mechanisms (e.g., mediators and moderators) operate in the relationship; (2) from methodological perspective, i.e., how other methodologies might be pursued in future empirical studies which might enhance the understanding of the supervisors' leadership and employees' employability which consequently represents a valuable contribution to employability literature.

To enhance theoretical knowledge within the leadership-employability relationship, findings suggest little research on the influence of other leadership concepts, such as relational (Clarke, 2018), authentic (Avolio et al., 2004) and empowering (Cheong et al., 2019) leadership, on employees' employability. Relational leadership is a social-process leadership style that stresses the role played by social interactions, which are dominated by mutual respect and trust between supervisors and employees (Clarke, 2018). Carifio (2010) argues that relational leadership includes 5 attributes—inclusive, empowering, caring, ethical, and vision and intuition—which might enhance employability. Authentic leadership (Avolio et al., 2004) is associates with followers' motivation and engagement enhancement, which subsequently foster employees' work outcomes (e.g., performance). Cheong et al. (2019) argue that empowering leadership relates closely to transformational and process leadership that provides relational support by, for example, fostering employees' autonomy. These leadership styles might operate as a job resource, enhancing employability. Therefore, we suggest further research directions by answer research questions such as: How will supervisors' leadership's (e.g., relational, authentic and empowering leadership) influence employees' employability? For instance, will supervisors' relational leadership enhance employees' employability more strongly, compared with authentic or empowering leadership? Moreover, despite the growing literature on collective forms of leadership such as shared leadership (Sweeney et al., 2019) which also may influence the employee-employer relationship in terms of employees' employability enhancement, we propose further research guided by the research question: How will shared leadership influences employees' employability?

Furthermore, the current systematic literature review uses an input-based approach of employability (Vanhercke et al., 2014), focusing on “the subjective perception held by an employee (or by his or her supervisor) about his or her possibilities in terms of competences, to obtain and maintain work” (Van der Heijden et al., 2018, p. 237), which is a limitation of this paper. Namely, according to De Lange et al. (2021), employability operationalizations can be categorized as “input- or competence-based” (p. 1) or “output- or labor market-based” (p. 1). In addition to this SLR, we therefore suggest to examine in future research how “output- or labor market-based” employability operates within the leadership-employability relationship.

We found that mechanisms such as mediators (e.g., work-related flow; Van der Heijden and Bakker, 2011) and moderators, for instance proactive personality (Wang et al., 2019), operate as mechanisms in the leadership-employability relationship. To explore further how supervisor leadership influences employee employability, we propose including other mediators and moderators as a starting point. We suggest assessing whether mediators, such as mutual respect and trust (Clarke, 2018), hope (Avolio et al., 2004), and psychological empowerment (Amundsen and Martinsen, 2015), operate in the relationship. More insights are also needed regarding whether moderators, such as a leader's gender (Cheong et al., 2019), influence the relationship. Thus, for this, we propose to answer, for instance, the research questions: Will mutual respect and trust, hope, and psychological empowerment mediate the leadership-employability relationship? And, how will leader gender moderate the leadership-employability relationship?

From a methodological perspective, cross-sectional designs dominated this review, which suggests validity concerns such as common method bias and causal inference (Rindfleisch et al., 2008). We suggest therefore that longitudinal designs with at least 3 waves of data collection, such as experiments (e.g., pretest-posttest, control-group designs) and multi-level designs, are needed to increase validity by assessing causal relationships between leadership and employability (Setia, 2016). Researchers should use multisource data (e.g., two-level data structures) among employees and immediate supervisors who work in under-researched contexts, such as healthcare and SME. Therefore, we propose to conduct research in new under-researched contexts, such as healthcare and SME, especially because outcomes of the supervisors' leadership—employability relationship are influenced by context effects (Oc, 2018).

### 4.2. Practical implications

This review offers several implications that inform policy, practice, and research in management and organization studies (Denyer and Tranfield, 2009), particularly related to employability and leadership. Findings demonstrate the possibilities of supervisor leadership as a job resource that enhances employability, contributing to employees' subjective career success. Employees are thus able to cope with rapidly changing jobs (Van der Heijden et al., 2018) and are less likely to develop feelings of job insecurity and panic in reaction to change because they are confident that they can pursue employment both inside or outside of the current organization if necessary (De Cuyper and De Witte, 2011). Policymakers should facilitate conditions that enhance supervisors' leadership as a job resource (e.g., contextual variable), which subsequently fosters employees' employability (i.e., a personal variable). From an organizational perspective, policymakers are able to foster supervisors' transformational leadership, for example, by facilitating training such as workshops (Bass and Avolio, 1990a), or feedback (Kelloway et al., 2000).

Supervisors should focus on the quality of LMX, being aware of the influence of shared self-identities and personal values between leaders and followers, which subsequently enhance the quality of relationships (Jackson and Johnson, 2012). Organizations should be aware of the role supervisors play as delegates of the organization, facilitating job resources such as perceived supervisor support. Supervisors should thus pay greater attention to providing job resources such as enhancing work experiences by offering informal social learning opportunities that foster employability (Froehlich et al., 2019). Among supervisors, insights suggest opportunities to enhance employability by creating a supportive work context with fit-for-purpose job resources. This is an important finding for policymakers, researchers, and supervisors.

### 4.3. Limitations

This review's methodology was crucial to assessing the current state of scientific research (Snyder, 2019), but it has some limitations. This review was influenced by several types of reporting biases, such as publication, location, and language biases (Higgins et al., 2019). Higgins et al. (2019) argue that publication bias, regardless of the expertise of the researcher, derives from whether articles get published. We are unable to review research findings in unpublished articles, which might influence results. Regarding location bias, we were restricted to using the university's interface. Thus, when identifying articles that met the inclusion criteria, we were restricted by access to a limited number of databases that are connected to the university's interface. Such restrictions determine access to articles that might influence findings. Language biases also affected results. Our inclusion criteria included peer-reviewed journal articles, conference articles, books, and book chapters published in English and Dutch. Due to a lack of resources to translate languages unknown to the researchers, findings were restricted to both languages, which might have influenced findings. We did not assess the quality of the methodologies used in the articles, which is also a limitation. Thus, the quality of the methodologies affected the quality of this review. When conducting a systematic review, the number of authors affects the review's quality. Two of the three authors independently identified, selected, and reviewed the articles used during the review, but a greater number of authors might have enhanced its quality. Despite the growing literature on collective forms of leadership such as shared leadership (Sweeney et al., 2019) we focus exclusively on the individual leadership-employability relationship which is a limitation of this paper. Finally, we exclusively look at “input- or competence-based” (De Lange et al., 2021, p. 1) employability, consequently we not search for “output- or labor market-based” (p. 1) employability, which is a restriction and thus a limitation of this paper.

## Data availability statement

The raw data supporting the conclusions of this article will be made available by the authors, without undue reservation.

## Author contributions

JH, AV, and JS presented the main idea of the manuscript. JH wrote the first draft of the manuscript, and AV and JS helped in the revision of the manuscript. All authors approved it for publication.

## Appendix

**Table A1:** Full list of searched major research databases using university's interface—January 2022 to February 2022.

• Academic Search Premier (EBSCO) • ACM Digital Library • AIS (Association for Information Systems) • APA PsycArticles (EBSCO) • APA PsycInfo (EBSCO) • Ars Aequi • Beeld en Geluid op school • Business Source Complete (EBSCO) • Cambridge Journals • CINAHL (EBSCO) • Cochrane Library • Company.info • Delpher • DOAJ – Directory of Open Access Journals • EBSCO Host (EBSCO) • Electronic Journals Service (EBSCO) • Embase on OvidSP • Emerald Insight [management] • ERIC EBSCO • Europeana • Europeana Newspapers • Factset • Google Scholar • Greenfile (EBSCO) • HeinOnline • IEEE Digital Library • Jesuit Online Bibliography • JSTOR • Kluwer Navigator • Lecture Notes in Computer Science • Legal Intelligence • Library Information & Technology Abstracts (EBSCO) • LibSearch: discovery tool UM • LiteRom (Nederlandstalig) • Max Planck Encyclopedias of International Law (MPIL) • Medline (EBSCO) • Wiley Online Library • Worldcat	• MUSE Humanities Collection • NARCIS • Nature • NDFR (Nederlandse Documentatie Fiscaal Recht) • Nederlandse historische parlementaire documenten • Nexis Uni (Lexis Nexis) • NLFiscaal • OA journal browser • Open Book Publishers • OpMaat Premium Plus • Overheid.nl • Oxford Historical Treaties (OHT) • Oxford International Organizations Database (OXIO) • Oxford Reports on International Law (ORIL) • Oxford Scholarly Authorities on International Law (OSAIL) • Oxford University Press • Psychology and Behavioral Sciences Collection (EBSCO) • Pubmed • Regional Business News (EBSCO) • Sage Business Cases • Sage Journals Online • Science • Sciencedirect (Elsevier) • SpringerLink • Strada Lex • Taylor & Francis Online • UN-iLibrary • Van Dale online woordenboeken • Web of Science • Westlaw UK • WestlawNext
